# Diffusion tensor MR imaging characteristics of cerebral white matter development in fetal pigs

**DOI:** 10.1186/s12880-017-0205-9

**Published:** 2017-08-22

**Authors:** Wenxu Qi, Song Gao, Caixia Liu, Gongyu Lan, Xue Yang, Qiyong Guo

**Affiliations:** 1 Department of Radiology, Shengjing Hospital, China Medical University, Shenyang, 110004 People’s Republic of China; 2 Morphology Teaching and Reasearch Section, Liaoning Vocational College of Medcine, Shenyang, 110100 People’s Republic of China; 3 Department of Obstetrics and Gynecology, Shengjing Hospital, China Medical University, Shenyang, 110004 People’s Republic of China

## Abstract

**Background:**

The purpose of this study was to investigate the anisotropic features of fetal pig cerebral white matter (WM) development by magnetic resonance diffusion tensor imaging, and to evaluate the developmental status of cerebral WM in different anatomical sites at different times.

**Methods:**

Fetal pigs were divided into three groups according to gestational age: E69 (*n* = 8), E85 (*n* = 11), and E114 (*n* = 6). All pigs were subjected to conventional magnetic resonance imaging (MRI) and diffusion tensor imaging using a GE Signa 3.0 T MRI system (GE Healthcare, Sunnyvale, CA, USA). Fractional anisotropy (FA) was measured in deep WM structures and peripheral WM regions. After the MRI scans,the animals were sacrificed and pathology sections were prepared for hematoxylin & eosin (HE) staining and luxol fast blue (LFB) staining. Data were statistically analyzed with SPSS version 16.0 (SPSS, Chicago, IL, USA). A P-value < 0.05 was considered statistically significant. Mean FA values for each subject region of interest (ROI), and deep and peripheral WM at different gestational ages were calculated, respectively, and were plotted against gestational age with linear correlation statistical analyses. The differences of data were analyzed with univariate ANOVA analyses.

**Results:**

There were no significant differences in FAs between the right and left hemispheres. Differences were observed between peripheral WM and deep WM in fetal brains. A significant FA growth with increased gestational age was found when comparing E85 group and E114 group. There was no difference in the FA value of deep WM between the E69 group and E85 group. The HE staining and LFB staining of fetal cerebral WM showed that the development from the E69 group to the E85 group, and the E85 group to the E114 group corresponded with myelin gliosis and myelination, respectively.

**Conclusions:**

FA values can be used to quantify anisotropy of the different cerebral WM areas. FA values did not change significantly between 1/2 way and 3/4 of the way through gestation but was then increased dramatically at term, which could be explained by myelin gliosis and myelination ,respectively.

## Background

From a simple tubular structure to a mature organ with complete function, the development and evolution of fetal brain is precise and complicated. White matter (WM) development of the intrauterine prenatal fetal brain is closely associated with a variety of nervous and mental diseases in the neonatal phase, early childhood, adolescence, and adulthood [1–10]. By studying and clarifying the intrauterine developmental patterns of cerebral WM before birth, and deciphering the anatomical and microstructural characteristics of fetal brain at different stages of development, we can not only analyze the procedures and steps of fetal brain developmental processes, but also study brain diseases related to brain development. Diseases such as perinatal brain injury and neonatal hypoxic-ischemic encephalopathy are closely related to cerebral white matter (WM) development [2]. Pigs are the standard animal model for studying neonatal hypoxic-ischemic encephalopathy (HIE) [11–14]. However, the mechanisms of normal fetal cerebral WM development have not been reported.

Diffusion tensor imaging (DTI) can quantitatively determine the parameters related to the movement direction of water molecules in the cerebral WM. DTI can not only quantitatively analyze the microstructure of cerebral WM, but also has the advantages of three-dimensional imaging of the cerebral WM fiber [15].

The current study utilized conventional MRI T2 structural imaging and DTI to measure the various specific characteristic FA values of different anatomical parts of the cerebral WM in fetal and neonatal pig brain, and used HE staining and LFB (Luxol Fast Blue) myelin staining to study the developmental changes in cerebral WM tissues, in order to determine the correlation between imaging and histology. The study allows preliminary exploration of the intrauterine developmental rules of pig cerebral WM at different stages.

Intrauterine prenatal fetal cerebral WM development is closely related to a variety of neurological and psychiatric diseases at the neonatal phase, early childhood, adolescence, and adulthood [1–10]. To study and clarify the developmental patterns of cerebral WM in the uterus before birth, and to clarify the anatomical and microstructure characteristics of fetal brain during different stages of development, we can not only analyze the procedures and steps of fetal brain developmental processes, but also study the brain diseases related to development. Diseases such as perinatal brain injury and neonatal hypoxic-ischemic encephalopathy are closely related to cerebral WM development [2].

The pig is the standard animal model for studying neonatal hypoxic-ischemic encephalopathy (HIE) [11–14]. However, studies investigating the normal fetal cerebral WM development have not been reported.

The current study utilized conventional MRI T2 structural imaging and DTI to measure the various specific characteristics FA values of different anatomical parts of the cerebral WM in fetal and neonatal pig brain, and used HE staining and FLB myelin staining to study the developmental changes in cerebral WM tissue, to determine the correlation between imaging and histology, thus allowing a preliminary exploration of the intrauterine developmental rules of pig cerebral WM at different stages.

## Methods

### Animal preparation

This study was conducted on the approval of Ethical Committee at Shengjing Hospital, China Medical University (Permit Number:2014PS153K). Through caesarean section, eight fetal pigs with gestational age of 69 days, and 11 fetal pigs with gestational age of 85 days were obtained from healthy pregnant pigs. Another six neonatal pigs from the same mother pig, with gestational age of 114 days, were also included in the current study. All the pigs were divided into three groups based on their gestational age, which were named as the E69 group, E85 group, and E114 group.

### MRI scanning

MRI scanning was carried out using a 3.0-T MR system (Signa Excite HD; GE Medical Systems, Milwaukee, Wis), with rat coil (5 cm in diameter) used for the E69 and E85 groups, and knee joint coil (15 cm in diameter) used for the E114 group. The conventional T2WI scan parameters were: TR5000 ms, TE80 ms, with layer thickness of 2 mm and interval of 0.3 mm. The SE-EPI sequence was utilized for DTI examination with scanning parameters as follows: TR8000 ms, TE100 ms; scan matrix: 128*128; Field of View: 4–6 cm; layer thickness of 2 mm, and interval of 0.3 mm. Scanning was carried out twice with a diffusion weighting coefficient b value of 0/600 s/mm2 and a gradient field intensity applied at 25 directions. The scanning time was about 12 min.

### Specimen preparation

After the MRI examination, the animals were sacrificed immediately with the whole brain quickly collected, then immersed, and fixed in 4% formalin solution. After 72-h fixation, the right and left hemispheres of the specimen were separated, followed by cross-sectional sampling, then embedded in paraffin and sliced.

### Regular HE staining

The morphology, quantity and the density of the glia cells, and the density of nervous fibers were evaluated by HE staining of sections. The neurons and glial cells were counted in highpower field (×200) .7 anatomic sites of the deep brain white matter and 4 anatomical sites of superficial brain white matter were selected for each of the brain specimens.

### LFB myelin specific staining

The method for LFB staining was: (1) Paraffin slice and alcohol dehydration; (2) staining with LFB, and incubation overnight with 1% LFB dye at 60 °C; (3) differential fixation with 70% alcohol immersion and 0.05% lithium carbonate; (4) washing in water; (5) HE counterstaining; and (6) mounting. The myelin sheaths stained blue using this method.

### Post-processing and analysis of the images

#### Images with conventional scanning

The T2WI image obtained by conventional scanning was analyzed by two experienced neuroradiologists using a double-blind approach. The semiquantitative maturity evaluation score method was as previously described [16]. Two experienced clinicians evaluated the images separately, with further discussions in cases of differing opinions.

#### DTI results

Data processing was carried out with GE Workstation Function Tool software (GE Healthcare). Regions of interest (ROIs) in the T2WI map, Colored orientation map, Average DC map and FA map were defined by two experienced radiologists who were not familiar with the gestational age of the study animals, with the FA values of each ROI measured. The ROIs are outlined on T2WI maps,also can be superimposed on the Colored orientation maps, Average DC maps and FA maps at the same time on GE Functool station.

**Fig. 1:**
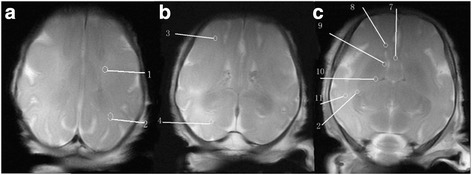
Fetal brain graph of interest region (from 1 to 12 were the corona radiate, parietal lobe white matter, frontal lobe white matter, occipital lobe white matter, corpus callosum genu, periventricular white matter, the anterior limb internal capsule, white matter of temporal lobe, optic radiation). (**a**) the level of semioval center (**b**) the level of lateral ventricle (**c**) the level of basal nuclei

The measuring method was the size of the ROI at a point region of 1 mm2, which was moved within the anatomical position to obtain the highest FA value. The selected ROIs included deep cerebral WM including the anterior limb internal capsule (ALIC), the posterior limb internal capsule (PLIC), the genu corporis callosi (GCC), the splenium corporis callosi (SCC), the periventricular white matter (PV), the optic radiation (OR), the corona radiate (CR), and the superficial white matter regions including the frontal lobe (FL), temporal lobe (TL), parietal lobe (PL), and occipital lobe (OL), as shown in Fig. 1. Measurements were of both sides of the brain.

#### Statistical analyses

SPSS version 15 (SPSS) was used for data analyses, and *P* < 0.05 was considered statistically significant. All data were expressed as mean ± standard deviation (M ± SD), with paired t-tests used to analyze whether there was a significant difference in the FA values of the same site between the left and right hemispheres. Spearman’s rank correlation analysis was used to analyze the correlation coefficient of the gestational age and FA values in different parts of the brain. The differences in FA values among different parts of the brain, or at different gestational ages, were analyzed using univariate ANOVA. The mean values of FA in the deep cerebral WM and the superficial cerebral WM were calculated. Spearman’s rank correlation analyses were utilized to determine the correlation coefficient between the FA value of the fetal cerebral WM and the gestational age. The differences between FA values and the number of neurons and glial cells of the deep and superficial cerebral WM, or at different gestational ages, were analyzed using univariate ANOVA.

## Results

### Morphological changes

**Fig. 2:**
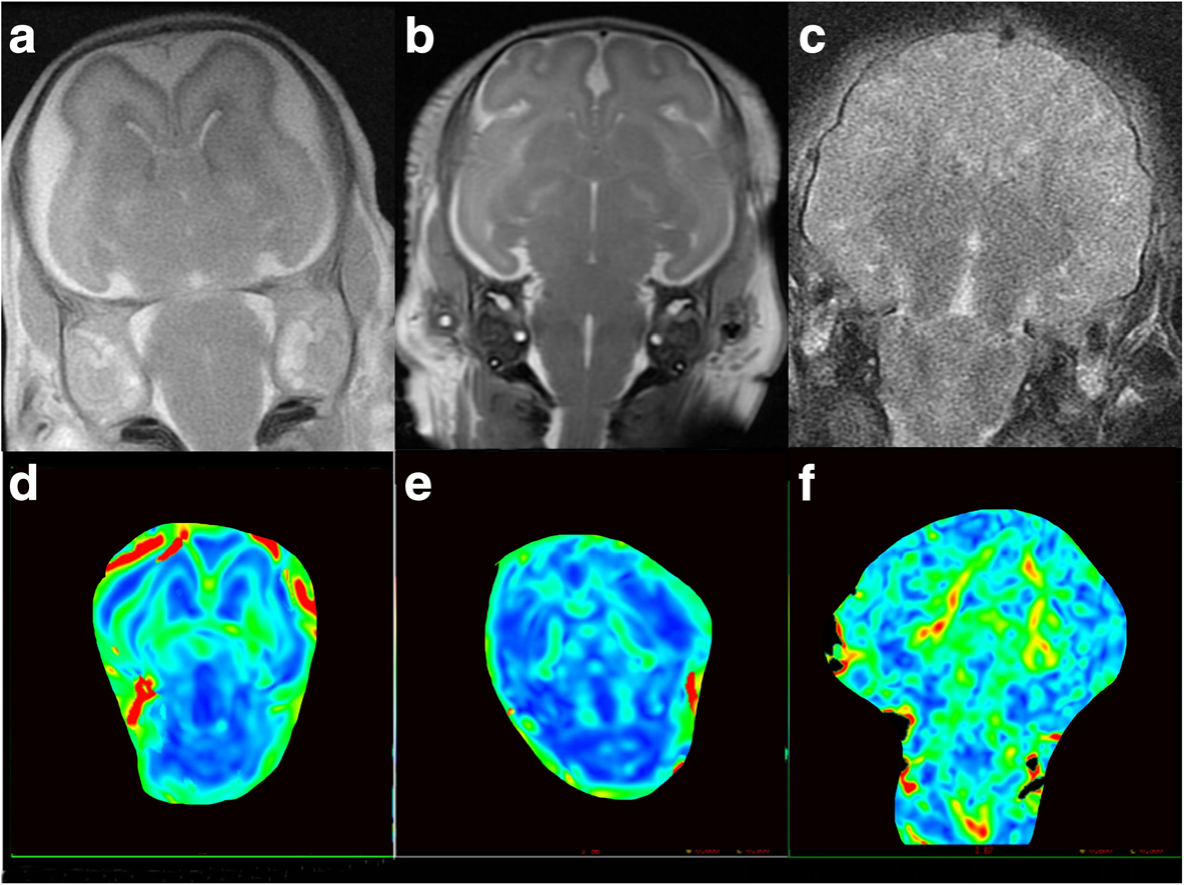
Basal ganglia T2WI map (**a**-**c**), FA (**d**-**f**) (from the left and right are gestational age 69, 85, 114 days respectively) No myelination changes were observed in the E69 group (**a**). In the E85 group, low punctate signals indicated myelination in the medulla, caudex cerebri, and at the back of pons in T2WI images, with no myelination in the PLIC and lateral parts of the thalamus (**b**). In the E114 group, a high signal was observed at the spinal cord, extending from the medulla oblongata and dorsal pons, through the medial lemniscus to the cerebral peduncle, thalamic ventral lateral, PLIC, and central part of the corona radiata (**c**)

No myelination changes were observed in the E69 group (Fig. 2), and were equivalent to the normal human levels of a gestational age younger than 20 weeks. In the E85 group, low punctate signals indicated myelination in the medulla, caudex cerebri, and at the back of pons in T2WI images, with no myelination in the PLIC and lateral parts of the thalamus. This was equivalent to the human level at 27–35 weeks of gestational age. In the E114 group, a low signal was observed at the spinal cord, extending from the medulla oblongata and dorsal pons, through the medial lemniscus to the cerebral peduncle, thalamic ventral lateral, PLIC, and central part of the corona radiata, consistent with the human level of neonates at 40 weeks of gestational age.

**Fig. 3:**
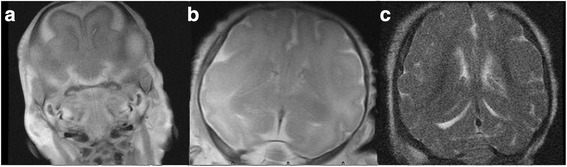
Germinal matrix T2WI map (**a**-**c**) (from the left and right are gestational age 69, 85, 114 days respectively) the E69 group had a low signal in the germinal matrix along the anterior horn, thalamic tail groove, and the posterior horn (**a**). The E85 group had a low signal corresponding to the anterior horn and thalamic tail groove, but not the posterior horn (**b**). In the E114 group, no germinal matrix was observed (**c**)

In the germinal matrix (Fig. 3), the E69 group had a low signal in the germinal matrix along the anterior horn, thalamic tail groove, and the posterior horn. The E85 group had a low signal corresponding to the anterior horn and thalamic tail groove, but not the posterior horn, which was equivalent to the human level at 26 weeks of gestational age. In the E114 group, no germinal matrix was observed, corresponding with the human level at older than 34 weeks.

**Fig. 4:**
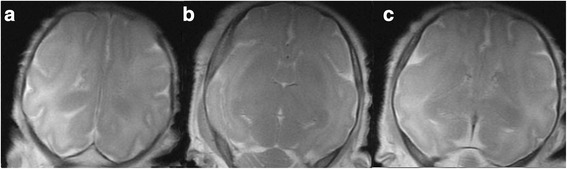
Cerebral sulcus development T2WI map (**a**-**c**) (from the left and right are gestational age 69, 85, 114 days respectively) In the E69 group, smooth insula with no anterior or posterior temporal sulcus, visible hemisphere cerebral sulcus, and a lateral cerebral fissure separating the frontotemporal and parietal occipital sulcus (**a**). In the E85 group, a shallow insular sulcus and gyrus with shallow anterior and posterior temporal sulcus were observed. Fissura calcarina, sulcus ammonis, the anterior central gyrus, the central gyrus, and the posterior gyrus were observed, along with a shallow anterior and posterior frontal gyrus, and anterior and posterior temporal gyrus (**b**). In group E114, a deep insular sulcus and gyrus, and deepened anterior and posterior temporal sulci were observed, along with a deep anterior and posterior frontal gyrus, anterior and posterior temporal gyrus, and temporal occipital gyrus, as well as a visible frontotemporal gyrus. The lateral fissure of the brain was almost closed, with the insula covered (**c**)

Sulci and gyri (Fig. 4) in the E69 group showed smooth insula with no anterior or posterior temporal sulcus, visible hemisphere cerebral sulcus, and a lateral cerebral fissure separating the frontotemporal and parietal occipital sulcus. In the E85 group, a shallow insular sulcus and gyrus with shallow anterior and posterior temporal sulcus were observed. Fissura calcarina, sulcus ammonis, the anterior central gyrus, the central gyrus, and the posterior gyrus were observed, along with a shallow anterior and posterior frontal gyrus, and anterior and posterior temporal gyrus. In group E114, a deep insular sulcus and gyrus, and deepened anterior and posterior temporal sulci were observed, along with a deep anterior and posterior frontal gyrus, anterior and posterior temporal gyrus, and temporal occipital gyrus, as well as a visible frontotemporal gyrus. The lateral fissure of the brain was almost closed, with the insula covered.

The brain maturity scores of the E69 group, the E85 group, and the E114 group were 1 point, 6 points, and 11 points, respectively, and were approximately equivalent to the levels of the human fetus at 20, 27, and 40 weeks, respectively [16].

### DTI data

There were no significant differences in the FA values found between the left and right cerebral hemispheres at the same site using paired t-tests (*P* > 0.05). The average values were calculated for further analyses.

The average FA values of the deep cerebral white matter in the different gestational age groups, including ALIC, PLIC, GCC, SCC, PV, OR, and CR and in the superficial cerebral WM, including Frontal Lobe,Parietal Lobe, Temporal Lobe and Occipital Lobe (Table 1). The FA values of different parts of the brain at the same gestational age were different. For the E114 group, the regions with the highest to lowest FA values were the PLIC, ALIC, SCC, GCC, PV, OR, CR, the occipital, parietal, temporal, and the frontal lobe WM. For the E85 group, the FA values were (from highest to lowest): PLIC, SCC, PV, OR, ALIC, GCC, CR, occipital, parietal, frontal, and temporal lobe WM. For the E69, the FA values were (from highest to lowest): PLIC, PV, SCC, GCC, ALIC, CR, frontal, parietal, temporal, and occipital lobe WM. During the process of overall development, among the deep cerebral WM, the FA values of the PLIC and SCC were higher, while the value of the OR and CR were lower.

**Table 1:** The average FA value of different parts of different gestational age

	E69	E85	E114
ALIC	0.214 ± 0.004	0.218 ± 0.011	0.452 ± 0.010
PLIC	0.233 ± 0.008	0.234 ± 0.009	0.587 ± 0.021
GCC	0.216 ± 0.007	0.217 ± 0.032	0.385 ± 0.103
SCC	0.229 ± 0.011	0.234 ± 0.012	0.428 ± 0.014
PV	0.231 ± 0.012	0.231 ± 0.008	0.337 ± 0.023
OR	0.212 ± 0.009	0.220 ± 0.009	0.285 ± 0.011
CR	0.203 ± 0.003	0.206 ± 0.010	0.280 ± 0.015
Frontal Lobe	0.086 ± 0.014	0.090 ± 0.025	0.145 ± 0.026
Temporal Lobe	0.069 ± 0.014	0.078 ± 0.032	0.151 ± 0.022
Parietal Lobe	0.084 ± 0.014	0.097 ± 0.012	0.173 ± 0.015
Occipital Lobe	0.069 ± 0.007	0.118 ± 0.008	0.230 ± 0.006

Spearman’s rank correlation analyses showed that the FA value of each ROI was positively correlated with gestational age, and the correlation was statistically significant (*P* < 0.01) (Table 2).

**Table 2:** Correlation analysis between FA value and gestational age at different sites

	r	P
ALIC	0.734	0.000
PLIC	0.579	0.002
GCC	0.715	0.000
SCC	0.580	0.002
PV	0.568	0.002
OR	0.760	0.000
CR	0.605	0.001
Frontal Lobe	0.510	0.008
Temporal Lobe	0.581	0.002
Parietal Lobe	0.741	0.000
Occipital Lobe	0.929	0.000

Note: R is the correlation coefficient, *P* < 0.05 has statistical significance

The univariate ANOVA analyses of the differences among the FA values of various regions at different gestational ages showed no statistically significant difference in the deep cerebral WM between the E69 group and E85 group, except for the ALIC and SCC (*P* > 0.05). There were no significant differences between the WM of the frontal and temporal lobes (*P* > 0.05). The average FA values of the deep cerebral WM were higher than those of the peripheral cerebral WM (*P* < 0.05) (Table 3).

**Table 3:** Deep brain WM and Superficial brain WM data table

	Deep brain WM		Superficial brain WM		T	P
	Average value	Standard deviation	Average value	Standard deviation		
E69	0.220	0.004	0.077	0.005	57.703	0.000
E85	0.223	0.006	0.095	0.014	25.216	0.000
E114	0.393	0.03	0.175	0.007	16.439	0.000
r	0.715		0.833			
P	0.000		0.000			

Note: R is the correlation coefficient, T is the single factor variance analysis

Spearman’s rank correlation analyses showed that the FA value of each ROI was positively correlated with gestational age, and the correlation was statistically significant (*P* < 0.01) (Table 3). The FA values of the anatomical regions were gradually increased with the gestational age, but at different speeds. The increasing change at the first stage, from the second trimester to late pregnancy, was slow (the deep and peripheral cerebral WM increases were 1.36% and 23.92%, respectively). The increase at the second stage, from the late pregnancy to neonatal stage, was greater (the deep and peripheral cerebral WM increases were 76.2% and 83.2%, respectively).

Univariate ANOVA analyses for the differences in FA values of different parts or different gestational ages showed no significant differences in the deep cerebral WM, except between that of the E69 group and the E85 group (*P* > 0.05).

### Histological examination of the pig fetal brain tissue

#### HE staining of the cerebral white matter

**Fig. 5:**
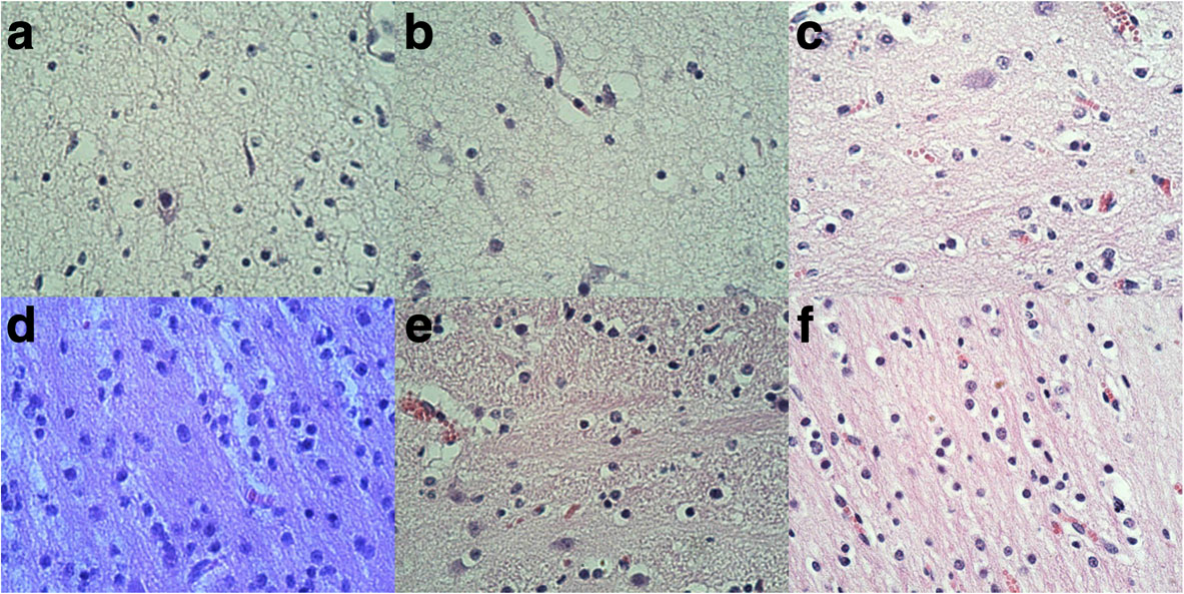
HE staining 200 times light microscope (from left to right frontal lobe, anterior parts) (**a**, **b**: E69 group; group **c** and **d**:E85; **e**, **f**: E114 group) T

HE staining and optical microscopy images of the fetal pig brain at different gestational ages (Fig. 5) demonstrated that the superficial WM neurons in the E69 group were immature with sparse nerve fibers and processes. The deep WM showed pink staining of the cytoplasm, with more mature neurons and sparse nerve fibers.

The nerve fibers of the E85 group were not densely clustered, the density of nerve fibers was not significantly different from that of the E69 group, but had significantly increased neurons. Compared with the E69 group, neurons in the deep cerebral WM of the E85 group were more densely clustered, mature, and close to the normal status, with larger numbers and densities of glia cells, more concentrated neuronal fibers, and increased numbers and lengths of cellular processes (axons or dendrites).

The morphology and numbers of the superficial WM neurons in the E114 group were close to that of the E85 group, but with denser nerve fibers. The morphology of the deep brain WM neurons in the E114 group was not significantly changed compared with the previous stage, but the nerve fibers were thicker and the glial cells became larger and more complicated, which corresponded with the beginning of myelination.

#### LFB staining of cerebral white matter

**Fig. 6:**
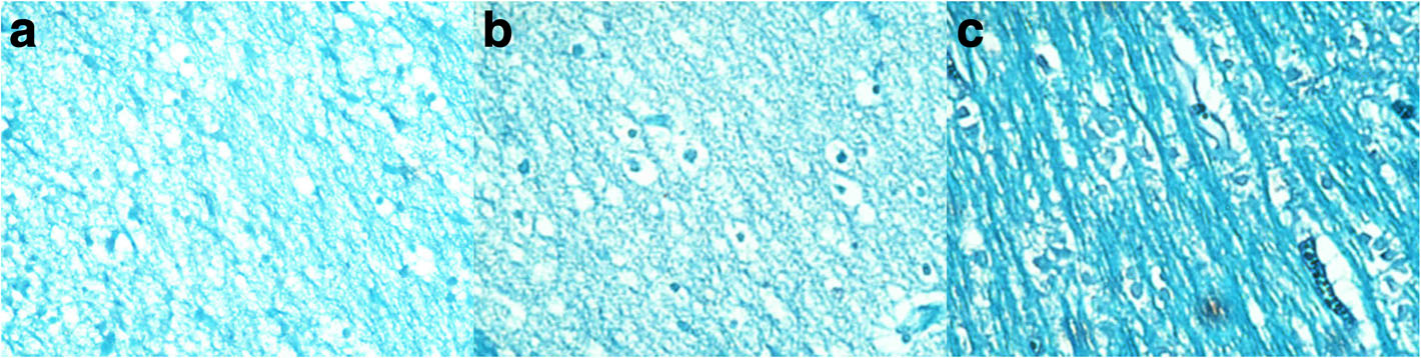
FLB staining 200 times light microscope (internal cerebral white matter: **a**: E69 group, **b**: E85 group, **c**: E114 group)

LFB staining of pig brain at different gestational ages (Fig. 6) showed bright blue staining of the myelin sheath visible under optical microscopy. Typical funicular distribution of blue staining was observed in the deep cerebral WM of the E114 group. No blue staining was observed in the deep and superficial cerebral WM of the E69 and E85 groups, or the superficial cerebral WM of the E114 group.

The number of neurons of the superficial WM in E69 group, E85 group, E114 group were 6.38 ± 3.045,13.73 ± 7.029,16.13 ± 9.993,respectively. The number of glial cells of the superficial WM in E69 group, E85 group, E114 group were 11.69 ± 3.505,61.98 ± 23.460,64.50 ± 26.203;respectively. The number of neurons of the deep WM in E69 group, E85 group, E114 group were 9.95 ± 4.952,18.81 ± 9.022,18.79 ± 8.446;respectively. The number of glial cells of the deep WM in E69 group, E85 group, E114 group were 13.50 ± 4.596,121.00 ± 40.811,116.84 ± 44.584,respectively.

Multiple groups means were compared with single factor analysis of variance, and the comparison among groups was performed with SNK method. Based on α = 0.05,There was no statistically significant difference in population mean of neurons and glial cells between the E85 group and E114 group, including the deep brain WM and superficial brain WM. In addition, there are significant statistically difference in population mean between any other two groups.

## Discussion

### DTI study of development animal brain

This study chose to examine the pig brains at gestational ages of 69, 85, and 114 days corresponding to the human gestational weeks of one half, three quarters, and full pregnancy, respectively. Earlier gestational ages, have smaller head diameter, cannot be studied because the unsuited magnetic resonance coil would affect the quality of the images. The data show that T2 images of conventional MRI demonstrated a degree of myelination, degeneration of the germinal matrix, and morphological changes of the brain sulcus roughly corresponding with the human fetal level of 20 weeks, 27 weeks, and 40 weeks. The FA values of the WM were consistent with the DTI study of the fixed primate fetal brain at 90–185 days of gestational age, and had almost the same FA values at all regions of the WM at a gestational age of 90 days, with no significant differences between the deep and superficial WM. At a gestational age of 185 days,the FA values of the WM were higher, especially for the myelinated WM such as the internal capsule [17], which is consistent with our observations and past studies.

There was also a study investigating the DTI changes in cat brain after birth. Although the cats were already born, the development of the myelin sheath was consistent with the intrauterine development of pig and primates; therefore, their FA value changes could also be used as references. The P0 samples showed diffusive WM with low FA values, which had slightly higher local FA values at areas with more concentrated neurons such as the corpus callosum and internal capsule. Along with the development (P35), LBS staining was used to detect myelination in deep cerebral WM, which showed major projection fibers such as primary visual and sensory motor fibers. In these regions, the FA values were highest, with increased FA values at the peripheral WM. These changes occurred at 1 month after birth, which was also consistent with the process we observed between the second trimesters to full pregnancy [18]. In addition, using the afterbirth development of rats as a reference for the study of human intrauterine development is also useful [3, 19, 20].

The rules of development and FA value changes of fetal cerebral WM DTI can reflect the anatomical and pathological processes of WM fibers, which is a great improvement in imaging methodology, and may also improve the field of WM fiber study [21]. Our results showed that the values of FA increased along with myelination development. Therefore, measuring values can be used as a noninvasive imaging quantitative marker for fetal cerebral WM maturation. The anisotropy of WM fibers is determined by macroscopic and microscopic conditions. First, the microstructural characteristics of the tissues, such as the diameter and density of the fibers, and the degree of the myelination, and then, the macrostructural characteristics of tissues, such as the interconnections among the fiber channels, can be determined. As summarized in previous studies, the histological development related to the rapid changes of FA values of the cerebral WM include increased numbers and densities of neuronal axons, enhanced phosphorylation of nerve fibers, increases in number and maturity of myelin sheaths of nerve fibers, and the expression of myelin basic protein [22, 23].

### The differences between the deep WM and the peripheral WM

The current study demonstrated that at various gestational ages, the FA values of the deep cerebral WM in fetal brain were significantly higher than in peripheral WM. Combined with histological examination results, a possible mechanism could be suggested: 1) In terms of axonal fiber arrangement, although the extent of development of the WM in this study is very immature [24], the structure of the deep cerebral WM is significantly more complete and complicated than the superficial WM, where the axons form parallel, compact, and bundled structures during the second trimester (15 to 28 weeks) [25, 26]. At the same time, the superficial WM of the cortex is very sparse, with scattered axon fibers in the WM. 2) Myelination of the deep cerebral WM starts from the second trimester, and reaches the PLIC at a gestational age of 35 weeks (the latest age evaluated in this study), and can expand to the main deep cerebral WM at the central part, such as in the center of the half oval. The superficial cerebral WM is mainly composed of tissues under the cortex that will develop into the far-end joint, contacting and projecting WM fiber to connect the two hemispheres of the cortex, but will not undergo myelination [22, 27]. 3) Regarding the direction of projections, the direction of the deep cerebral WM is more consistent with the unitary direction of the projection, union, and contact of nerve fibers, with almost no distortion and more concentrated directions. While the superficial part of the cerebral WM is an extension of the deep cerebral WM into the cortex, the direction is more scattered. 4) Regarding the shaft radius, the radius of the scattered fiber is obviously smaller than the radius of the central axis. 5) The deep cerebral WM is also known as dense WM, the structure of which is relatively dense. With dense neurons and lower water content, the FA values of the deep brain WM are high at various developmental phases. However, the superficial cerebral WM neurons are sparse and contain a large amount of extracellular fluid. A previous study [28] concluded that the increase of FA values in brain parenchyma was due to decreased water content in the brain and the convergence of macromolecular materials, which is also consistent with another report [29]. The results of this study showed that the developmental pattern of the cerebral WM is in the order of center to periphery, posterior to anterior, and bottom-up, which has been confirmed by additional studies [30].

### Changes of FA in the deepWM during development

The current study demonstrated the temporal variation of the deep cerebral WM in different fetal age groups. FA values did not change significantly between 1/2 way and 3/4 of the way through gestation but was then increased dramatically at term. These findings are consistent with in vivo observations of human intrauterine development [31] and the model based on the hypothesis of premature infant development [29, 32]. Intrauterine brain development studies of 23–38 weeks evaluated the changes in the corticospinal tract, optic radiation, and corpus callosum, while premature infant brain development studies evaluated the postnatal changes of the callosity body, cerebral peduncle, corticospinal tract, spinothalamic tract, internal capsule, radiation, inferior longitudinal fasciculus, and cingulum at 1–4 month after birth. It was found that “axonal organization”, “myelin gliosis”, and “myelination” corresponded with increased, unchanged, and increased FA values, respectively. The “axonal organization” manifested as scattered axons transformed into a coherent and clear bundle of nerves. The diffusion direction of water is gradually limited to the direction of the axon (from central to the periphery). The model of the water molecule diffusion direction increases. The “myelin gliosis”manifested as glial cells formed around neurons, which does not change the diffusion direction of the water molecules, and confirms the model of water molecular diffusion direction. During the process of “myelination”, the direction of water molecule diffusion is limited to radiation, that is, from peripheral to axons. The “myelin gliosis” is more specifically reflected in the corticospinal tract at 28.5–32.5 weeks, or at 26.3–34.8 weeks, the SCC at 25.6–35.4 weeks, and the GCC from 25 weeks to after birth (38 weeks), suggesting that FA values would not significantly change, consistent with the histological observations [33, 34]. The data correspond to the lack of significant change from 1/2 to 3/4 pregnancy term in the deep cerebral WM, and the dramatic increase from 3/4- to full pregnancy observed in animal studies. The change of FA values during the period of “myelin gliosis” was not obvious, while the dramatic elevation of FA values during “myelination”in the premature infants and neonates has been reported in many studies [35–37]. Myelination is an important factor affecting the increase of FA values. The slow increase of FA values of the superficial cerebral WM may therefore be related to the microstructural development that limits water dispersion, including gradual densification of WM and lowered water content. The data and changes of FA values correspond with our histological findings, indicating that the FA values, reflecting the diffusion tensor anisotropy of magnetic resonance, can quantitatively reflect the level of the intrauterine developmental maturity of the fetal cerebral WM, and the changes of FA values could be used to divide cerebral WM development into different stages. From the perspective of guiding clinical practice, during “myelin gliosis”period, precursors of oligodendrocytes in the WM gradually develop into immature oligodendrocytes, which may correspond to the high-risk time frame (23–32 weeks) of periventricular leukomalacia (PVL). At this stage, the majority of oligodendrocytes is at the phase of advanced oligodendrocyte precursors, which is suspected to be a potential target of PVL [33, 34].

### Limitations

Limitations of the current study include: 1) Because the head size of the experimental animals did not match the rat coil, the study time period was set after mid pregnancy. 2) Due to experimental conditions, including limits such as field strength and coil, the quality of animal experimental images need to be improved. Different diameter of the magnetic resonance scan coils and different diameter of the animal heads, using the same scanning parameters may lead to relatively poor quality of T2WI. However, These features included degree of sulcation, extent of visualization of the germinal matrix, extent of myelination can be clearly distinguished,it does not affect the experimental results. 3) The size and location of ROIs have a great influence on the results of FA values. 4) Because the size of the animal’s head is small, the structure is not easy to identify, therefore there was less anatomical evaluation.

## Conclusions

In conclusion, we showed that FA values of DTI images reflect the anisotropy characteristics of the cerebral microstructural development, which can be used for quantitative analysis of the intrauterine developmental changes of cerebral WM in the fetal pig. The FA values of the deep cerebral WM were higher than that of the superficial cerebral WM, which histologically reflect the higher maturity of the deep cerebral WM when compared with the superficial WM. FA values did not change significantly between 1/2 way and 3/4 of the way through gestation, which were related to the myelin gliosis. However, it was then increased dramatically at term may be closely related to the myelination of nerve fibers.

## Abbreviations

ALIC: The anterior limb internal capsule
CR: The corona radiate
DTI: Diffusion tensor imaging
FA: Fractional anisotropy
GCC: The genu corporis callosi
HE: Hematoxylin & eosin
HIE: Hypoxic-ischemic encephalopathy
LFB: Luxol fast blue
MRI: Magnetic resonance imaging
OR: The optic radiation
PLIC: The posterior limb of the internal capsule
PV: The periventricular white matter
ROI: Region of interest
SCC: The splenium corporis callosi
WM: White matter
